# Non-Coding RNAs Regulate the Resistance to Anti-EGFR Therapy in Colorectal Cancer

**DOI:** 10.3389/fonc.2021.801319

**Published:** 2022-01-17

**Authors:** Jinjin Chu, Xianzhu Fang, Zhonghou Sun, Linlin Gai, Wenqing Dai, Haibo Li, Xinyi Yan, Jinke Du, Lili Zhang, Lu Zhao, Donghua Xu, Shushan Yan

**Affiliations:** ^1^ Central Laboratory of the First Affiliated Hospital, Weifang Medical University, Weifang, China; ^2^ Department of Pathology and Pathophysiology, Weifang Medical University, Weifang, China; ^3^ Department of Pediatrics of the First Affiliated Hospital, Weifang Medical University, Weifang, China; ^4^ Department of Gastrointestinal and Anal Diseases Surgery of the Affiliated Hospital, Weifang Medical University, Weifang, China

**Keywords:** miRNA, lncRNA, circRNA, CRC, EGFR, drug resistance

## Abstract

Colorectal cancer (CRC) is the third prevalent cancer worldwide, the morbidity and mortality of which have been increasing in recent years. As molecular targeting agents, anti-epidermal growth factor receptor (EGFR) monoclonal antibodies (McAbs) have significantly increased the progression-free survival (PFS) and overall survival (OS) of metastatic CRC (mCRC) patients. Nevertheless, most patients are eventually resistant to anti-EGFR McAbs. With the intensive study of the mechanism of anti-EGFR drug resistance, a variety of biomarkers and pathways have been found to participate in CRC resistance to anti-EGFR therapy. More and more studies have implicated non-coding RNAs (ncRNAs) primarily including microRNAs (miRNAs), long non-coding RNAs (lncRNAs), and circular RNAs (circRNAs), are widely involved in tumorigenesis and tumor progression. They function as essential regulators controlling the expression and function of oncogenes. Increasing data have shown ncRNAs affect the resistance of molecular targeted drugs in CRC including anti-EGFR McAbs. In this paper, we have reviewed the advance in mechanisms of ncRNAs in regulating anti-EGFR McAbs therapy resistance in CRC. It provides insight into exploring ncRNAs as new molecular targets and prognostic markers for CRC.

## Introduction

**Figure 1:**
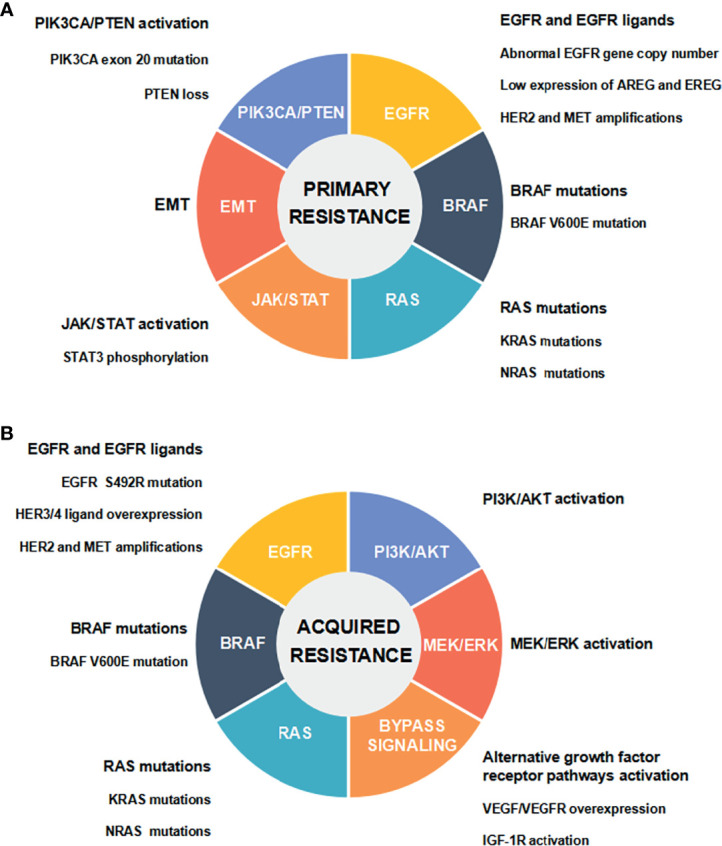
Mechanisms of anti-EGFR drug resistance in CRC. **(A)** Primary resistance mechanisms. **(B)** Acquired resistance mechanisms.

CRC is the third most frequent cancer worldwide. Global cancer statistics in 2020 has shown there are about 1.932 million new cases and 935,000 deaths of CRC worldwide, accounting for 10.0% of the total new cases of cancer and 9.4% of the total cancer-related deaths, respectively (1). Following lung cancer, CRC causes the second highest mortality in cancer patients worldwide (1). The therapeutic strategies for CRC mainly include surgery, chemotherapy, radiotherapy and targeted therapy. Currently, surgery and chemotherapy are still the preferred treatment options for CRC. Nevertheless, patients with metastatic CRC (mCRC) have a poor prognosis (2). The combined chemotherapy and molecular targeted drugs can noticeably increase the progression-free survival (PFS) and overall survival (OS) of mCRC patients (3). As molecular targeted drugs, cetuximab and panitumumab can directly target epidermal growth factor receptor (EGFR). Combined with chemotherapeutic drugs, they are applied to effectively treat mCRC patients carrying wild-type *RAS* and *BRAF* (4). Unfortunately, few patients with mCRC are sensitive to anti-EGFR treatment, and most responding patients usually develop resistance to the therapy (5). In recent years, a variety of biomarkers and pathways have been found to participate in regulating the resistance to anti-EGFR therapy, and thus affecting the therapeutic effect and reducing the survival rate of CRC patients (6). Some studies have suggested the potential resistance mechanisms in order to explore strategies for overcoming anti-EGFR resistance (5, 7, 8) **(** **Figure 1**).

EGFR is a kind of HER tyrosine kinase receptor, which is composed of extracellular ligand binding domain, transmembrane hydrophobic domain, and intracellular tyrosine kinase domain. EGFR is selectively activated by binding to epidermal growth factor (EGF) as one of the major ligands. EGFR transmits signals from cytoplasm to nucleus through RAS/RAF/MEK/ERK/MAPK, PI3K/PTEN/AKT/mTOR, and some other intracellular signaling pathways which participate in regulating cancer cell proliferation, invasion, and angiogenesis (9). Abnormal expression and activation of any signal molecules mentioned above may lead to primary (*de novo*) or acquired (secondary) resistance to anti-EGFR therapy in mCRC (5). Abnormal EGFR gene copy number, protein expression of EGFR ligands, HER2 and MET gene amplifications, and activation of EGFR downstream cascade signaling pathways [including the mutations of RAS/BRAF/PIK3CA, the loss of PTEN, STAT3 phosphorylation, and epithelial-mesenchymal transition (EMT)], have been demonstrated to be associated with the primary resistance to anti-EGFR therapy in CRC (5, 7, 8). It has been well documented that the acquired resistance is attributed to EGFR ectodomain mutation (S492R), genetic alterations in RAS/RAF and other downstream signaling molecules, and the activation of intracellular signaling pathways that are bypassing EGFR and mediated by IGF1R, HER2, MET, and VEGFR (5, 7, 8). Multiple genetic and nongenetic mechanisms drive resistance to anti-EGFR therapy in CRC, with a significant overlap in primary and acquired resistance (8) (**Figure 1**).

NcRNAs are a type of RNAs which have no protein-coding function. According to the length, they are divided into two classes: small non-coding RNAs (sncRNAs) with a length of 18-200 nt, and long noncoding RNAs (lncRNAs) with a length over 200 nt. NcRNAs are widely involved in cell proliferation, apoptosis, autophagy, EMT, and cell cycle progression (10–14). Accumulated studies have suggested ncRNAs play important roles in tumorigenesis, progression, and anti-EGFR monoclonal antibodies (McAbs) treatment resistance in CRC (15–21). In this review, we have focused on current progress in the underlying molecular mechanisms of ncRNAs in regulating the resistance to anti-EGFR therapy in CRC. We aim to fully explore the potentials of ncRNAs as novel molecular targets and prognostic markers for CRC.

## MiRNAs

### Biological Functions of MiRNAs

MiRNAs are single-stranded small ncRNAs with a length of 21-25 nt. The synthesis of miRNAs involves multiple biological steps. Firstly, primary miRNAs (pri-miRNAs) are encoded by DNA in the nucleus and transcribed by ribonucleic acid polymerase II. Secondly, long pri-miRNAs are processed by ribonuclease III Drosha, which produces precursor miRNAs (pre-miRNAs) with a length of 60-70 nt. Lastly, pre-miRNAs are cleaved into mature double-stranded miRNAs by ribonuclease III Dicer in the cytoplasm. Then, mature miRNAs participate in forming RNA-induced silencing complex (RISC) (22). MiRNAs induce messenger RNA (mRNA) degradation and translation repression by directly binding to the 3’-untranslated region (3’-UTR) of targeted mRNAs, and act as regulators at the post-transcriptional level during gene expression process (23). They are widely involved in cell proliferation, apoptosis, autophagy, and immune response (10, 11, 18, 21). Accumulated studies have suggested miRNAs participate in the pathogenesis of various diseases including cancers (24–28). MiRNAs act either as oncogenic miRNAs (onco-miRs) or tumor suppressive miRNAs (TS-miRs) with significant tissue- and organ-specificity (29, 30). Many studies have also found that miRNAs participate in regulating the drug resistance in CRC (31, 32). It has been demonstrated miR-31 negatively regulates breast cancer invasion and metastasis (33). However, it negatively regulates the expression of tumor suppressors and thus exerts oncogenic effects in lung cancer (34). In CRC, miR-31 has been documented to promote cancer progression by activating RAS signaling pathway and hypoxia inducible factor 1α (HIF-1α), respectively (35, 36). Taken together, miR-31 is involved in tumor progression and metastasis by serving as a TS-miR or an onco-miR in different malignancies. The diverse roles of miR-31 in cancer may be attributed to different types of cancer cells, specific targets, and other complicated factors. Further research is required to reveal its specific functions in CRC.

**Figure 2:**
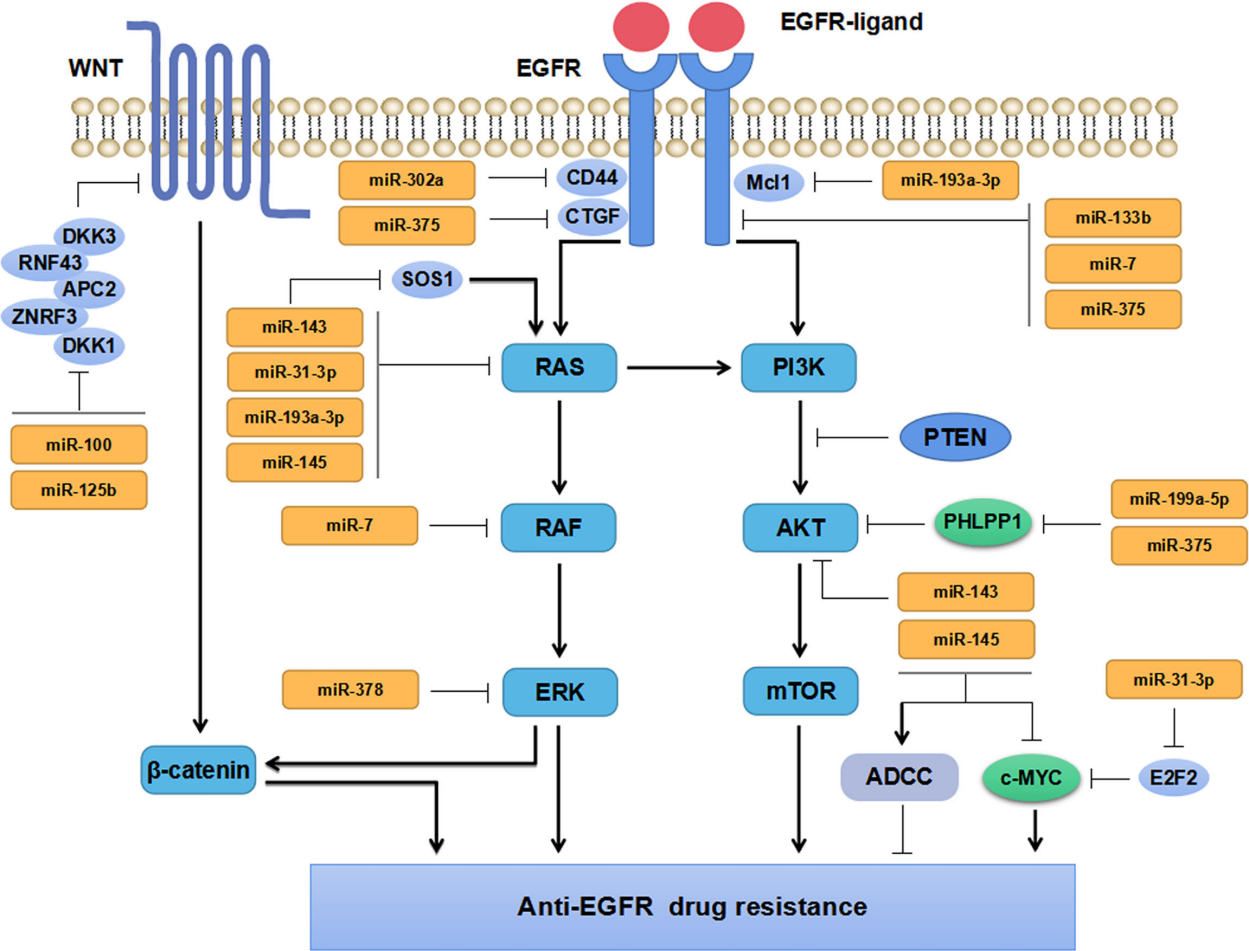
Mechanisms of miRNAs regulating anti-EGFR drug resistance in CRC.

MiRNAs, aberrantly expressed in tumor tissues and tumor cells, exert their tumor suppressive- and oncogenic-functions by regulating different targeted genes (**Figure 2**). When the expression levels of TS-miRs decrease, negative regulation on targeted genes weakens. Besides, increasing expression levels of onco-miRs promotes tumor development, metastasis, and drug resistance through down-regulating tumor suppressive genes.

### MiRNAs Regulate Drug Resistance of Anti-EGFR Therapy in CRC

MiRNAs regulates anti-EGFR drug resistance by directly targeting tumor-related genes involved in EGFR-related signaling pathways in CRC. Abnormal expression of miRNAs is commonly observed in anti-EGFR treatment-resistant CRC cells. Recent studies have shown that miRNAs may predict the prognosis and drug therapeutic efficacy of CRC patients (37–39). The latest studies regarding miRNAs and drug resistance to anti-EGFR therapy in CRC have been described in the following subsections and briefly summarized in **Table 1**.

**Table 1:** MiRNAs involved in anti-EGFR drugs resistance in CRC.

MiRNAs	Expression	Targets/Pathways	Drugs	References
MiR-133b	Down-regulated	EGFR pathway	Cetuximab	(40)
MiR-7	Down-regulated	EGFR/RAF pathway	Cetuximab	(41)
MiR-302a	Down-regulated	CD44/EGFR/RAS/MAPK pathway, CD44/EGFR/PI3K/AKT pathway	Cetuximab	(42)
MiR-143	Down-regulated	SOS1/RAS/ERK/MAPK pathway, AKT pathway	Cetuximab	(43)
		ADCC		(44)
		RAS-MAPK axis, c-MYC pathway		(45)
MiR-145	Down-regulated	ADCC	Cetuximab	(44)
		RAS-MAPK axis, c-MYC pathway		(45)
MiR-193a-3p	Down-regulated	KRAS/RAF/MEK/ERK pathway	Cetuximab	(46)
		Mcl1/EGFR/BRAF/MEK/MAPK pathway	Dabrafenib, Trametinib, Cetuximab	(47)
MiR-378	Down-regulated	ERK/MAPK pathway	Cetuximab	(48, 49)
MiR-31-3p	Up-regulated	RAS-MAPK axis, E2F2/c-MYC pathway	Cetuximab	(45)
MiR-100	Up-regulated	DKK1, ZNRF3/Wnt/β-catenin pathway	Cetuximab	(50)
MiR-125b	Up-regulated	ZNRF3, RNF43, DKK3, APC2/Wnt/β-catenin pathway	Cetuximab	(50)
MiR-199a-5p	Up-regulated	PHLPP1/AKT pathway	Cetuximab	(51)
MiR-375	Up-regulated	PHLPP1/AKT pathway	Cetuximab	(51)
	Down-regulated	CTGF/EGFR/PIK3CA/AKT pathway, EGFR/KRAS/BRAF/ERK1/2 pathway		(52)

### Impact of MiRNAs on EGFR Signaling Pathway

EGFR signaling pathway has been confirmed to be aberrantly activated in multiple malignant tumors, which is associated with tumor progression and prognosis. Increasing evidence has implicated miRNAs participate in regulating EGFR signaling pathway and play vital roles in anti-EGFR drug resistance in CRC (**Figure 2**). Zhou et al. have found miR-133b regulated cell proliferation and invasion in CRC by targeting EGFR (40). Moreover, the combination of miR-133b mimics and cetuximab can effectively suppress the proliferation and invasion of cetuximab-resistant CRC cells (40). Suto et al. have found miR-7 is involved in regulating the EGFR signaling pathway by down-regulating the expression of EGFR and RAF-1, which could inhibit CRC cells proliferation and reverse cetuximab resistance in CRC patients with mutant *KRAS* (41). Sun and the colleagues have found that miR-302a suppressed CRC metastasis by targeting nuclear factor I B (NFIB) and CD44 and decreasing the activation of NFIB/ITGA6 signaling pathway (42). MiR-302a has also been found to restore the response to cetuximab by inhibiting CD44-induced cancer stem cell (CSC)-like characteristics through EGFR-mediated MAPK and protein kinase B (AKT) signaling pathways (42). These studies have revealed that miRNAs can directly target EGFR (or RAF) in CRC cells, inhibit the activation of its downstream signaling pathways, and thus repress CRC cells growth and invasion. Besides, miR-100 and miR-125b have been found to cooperatively regulate the resistance to cetuximab in CRC through Wnt signaling pathway that has a cross-talk with EGFR pathway (50). MiRNAs are extensively involved in regulating the resistance to cetuximab. Accordingly, miRNAs might serve as markers for predicting anti-EGFR therapy in mCRC patients due to their regulatory effects on EGFR signaling pathway.

### Impact of MiRNAs on RAS Signaling Pathway

KRAS, a member of RAS family, has almost 40% mutation rate in CRC patients. KRAS mutations are predictive biomarkers for the treatment efficacy of anti-EGFR treatment and the outcome of patients with CRC (53). MiRNAs have been widely reported to regulate the therapeutic response and drug sensitivity of CRC patients through KRAS signaling pathway (43–45) (**Figure 2**).

Synthetic miR-143 (miR-143#12) inhibits KRAS signaling pathway activation and restores the sensitivity of cetuximab-resistant CRC cells by targeting the KRAS activating protein SOS1 (43). Overexpression of miR-143 or miR-145 can increase the sensitivity to cetuximab by enhancing cetuximab-mediated antibody-dependent cellular cytotoxicity (ADCC) in CRC cells (44). Strippoli et al. have demonstrated miR-31-3p, miR-143 and miR-145 are closely correlated with anti-EGFR treatment resistance in mCRC *via* regulating RAS-MAPK axis and c-MYC pathway (45). Moreover, miR-143 and miR-145 have been well established to exert tumor-suppressive effects and are beneficial for the efficacy of anti-EGFR treatment in CRC, whereas miR-31-3p comes to the opposite. It has been shown that the overexpression of miR-193a-3p can promote *BRAF*-mutant CRC cells apoptosis by inhibiting the expression of KRAS and myeloid cell leukemia-1 (Mcl1) through MAPK signal (47). As a tumor suppressor, miR-193a-3p promotes the efficacy of BRAF inhibitor dabrafenib (DAB) and MEK inhibitor trametinib (TRA), and enhances the anti-proliferative effect of combined therapy of DAB, TRA with cetuximab in CRC (47). A recent study has shown that 4-acetyl-antroquinonol B (4-AAQB) inhibits CRC cell proliferation and induces cell apoptosis by up-regulating miR-193a-3p, down-regulating KRAS and inhibiting the activation of KRAS signaling pathway. The combined treatment of 4-AAQB with cetuximab can make KRAS-mutant CRC cells resensitized to cetuximab (46). In addition to KRAS, miR-193a-3p acts on multiple signaling pathways and plays a tumor-suppressive role by regulating the expression of interleukin 17 receptor D (IL17RD) and erb-b2 receptor tyrosine kinase 4 (ERBB4) in CRC (54, 55). And lower expression of miR-193a-3p in CRC tissues predicts poorer PFS independently of the status of *BRAF* mutation (56). Accordingly, miR-193a-3p may serve as a prognostic biomarker. Its combination with molecular targeted drugs may be a novel therapeutic strategy for *BRAF*-mutant CRC. Weng et al. have reported that lauric acid can induce miR-378 expression and increase the sensitivity of *BRAF*- and *KRAS*-mutant CRC cells to cetuximab by inhibiting KRAS, BRAF, MEK, ERK1/2 protein expressions through the MAPK signaling pathway (48). In addition, they have found that eicosapentaenoic acid ethyl ester (EPA) can also increase the expression of miR-378 in *BRAF-* and *KRAS-*mutant CRC cells and resensitize *KRAS-*mutant CRC cells to cetuximab (49). Taken together, miRNAs play vital roles in regulating the therapeutic response and drug sensitivity of KRAS- or BRAF-mutant CRC through RAS signaling pathway. Potential miRNAs and key molecules in the RAS signaling pathway may serve as promising biomarkers for predicting the efficacy and drug resistance during the targeted therapy in CRC.

### Impact of MiRNAs on PI3K/AKT Signaling Pathway

The PI3K/AKT signaling pathway is widely involved in carcinogenesis and cancer progression. Aberrant activation of PI3K-AKT can promote CRC invasion and metastasis (57). It has been reported that miRNAs can directly target the PI3K/AKT signaling molecules or signaling pathway regulators, including numerous regulatory proteins (51, 52, 57–60) (**Figure 2**). MiR-375 and miR-199a-5p promote cetuximab resistance in CRC patients by repressing the expression of PH domain and leucine-rich repeat protein phosphatase 1 (PHLPP1) and positively regulating AKT signaling pathway (51). Nevertheless, some other studies have found miR-375 and miR-199a-5p inhibit CRC cells proliferation and invasion, suggesting their complicated functions in CRC (52, 58–60). It has been well documented that miR-375 suppressed CRC cell proliferation by targeting PIK3CA *via* the PI3K/AKT pathway (61), while miR-199a-5p inhibited CRC cell survival, proliferation, migration, and invasion by downregulating GCNT2 expression and inhibiting the AKT and ERK signal activation (62). Different roles of miR-375 and miR-199a-5p exerting in CRC, might be attributed to significant tumor heterogeneity among patients. Taken together, miRNAs regulate the progression and drug resistance of CRC by regulating tumor suppressors or oncogenes involved in various signaling pathways including PI3K/AKT signal. However, the precise mechanisms of miR-375 and miR-199a-5p underlying the resistance to anti-EGFR drugs in CRC warrant to be fully elucidated in more future research.

### Impact of MiRNAs on Tumor Immune Microenvironment

Tumor immune microenvironment is composed of a variety of cells, extracellular matrix and various signaling molecules (63). Imbalance of tumor immune microenvironment is essential for tumor growth, metastasis and prognosis (64). MiRNA-mediated regulation of tumor microenvironment (TME) has been demonstrated to affect cancer growth, angiogenesis, metastasis, and drug resistance exerting either antitumor or tumorigenic effects (65). For instance, a recent study has shown miR-34a promoted the expression of B7-H3 and TNF-α in tumor microenvironment and negatively regulated T cell-mediated immune response, which thus induced immunosuppression and immune escape in CRC (66). MiR-148a-3p and miR-448 respectively down-regulate the expression of calnexin (CANX) and indoleamine 2,3-dioxygenase 1 (IDO1), which enhances CD8^+^ T cell-mediated immune response in CRC (67, 68).

Cetuximab can induce ADCC by binding to EGFR on cancer cells and CD16 receptor on natural killer (NK) cells and dendritic cells (DCs) (69–71). It stimulates the production of proinflammatory cytokines, such as IFN-γ and TNF-α, and activates cytotoxic T cells in the TME, thereby exerting tumor immunosuppressive effects (69–71). It has been suggested that anti-EGFR therapy and immunotherapy have synergetic and complementary mechanisms. The combination of immune checkpoint inhibitors, chemotherapy with anti-EGFR McAbs in mCRC has shown an encouraging clinical outcome (72). Nevertheless, littler is known about the role of miRNAs in regulating tumor immune microenvironment and thus affecting anti-EGFR drugs resistance in CRC.

## LncRNAs

### Biological Functions of LncRNAs

LncRNAs are a type of ncRNAs over 200 nt in length. They are mainly formed by RNA polymerase II-catalyzed transcription typically containing a cleavable 3’ poly-A tail (73). According to genomic localization, lncRNAs are grouped into five classes: sense lncRNA, antisense lncRNA, intronic lncRNA, bidirectional lncRNA, and intergenic lncRNA (74). LncRNAs have low sequence conservation and high tissue and organ specificity. As competitive endogenous RNAs (ceRNAs), lncRNAs can directly sponge miRNAs and inhibit their expression. LncRNAs interact with DNA, RNA and protein, acting as regulators of gene expression at multiple levels and play roles in various cell processes, such as genomic imprinting, epigenetic regulation, transcriptional regulation, chromosome conformation, and cell cycle regulation (75). A great deal of data has suggested lncRNAs participate in the pathogenesis of various diseases, including cancer (75–78). Linc00152, SNHG1, SCARNA2, DLEU1 and XIST contribute to colorectal carcinogenesis, metastasis and prognosis of CRC (54, 79–83). In addition, a number of studies have implicated lncRNAs lead to associated with primary or acquired drug resistance in CRC, thereby reducing drug efficacy (84, 85). Nonetheless, the regulatory mechanisms of lncRNAs underlying anti-EGFR therapy resistance in CRC are not clear yet.

### LncRNAs Regulate Drug Resistance of Anti-EGFR Therapy in CRC

**Figure 3:**
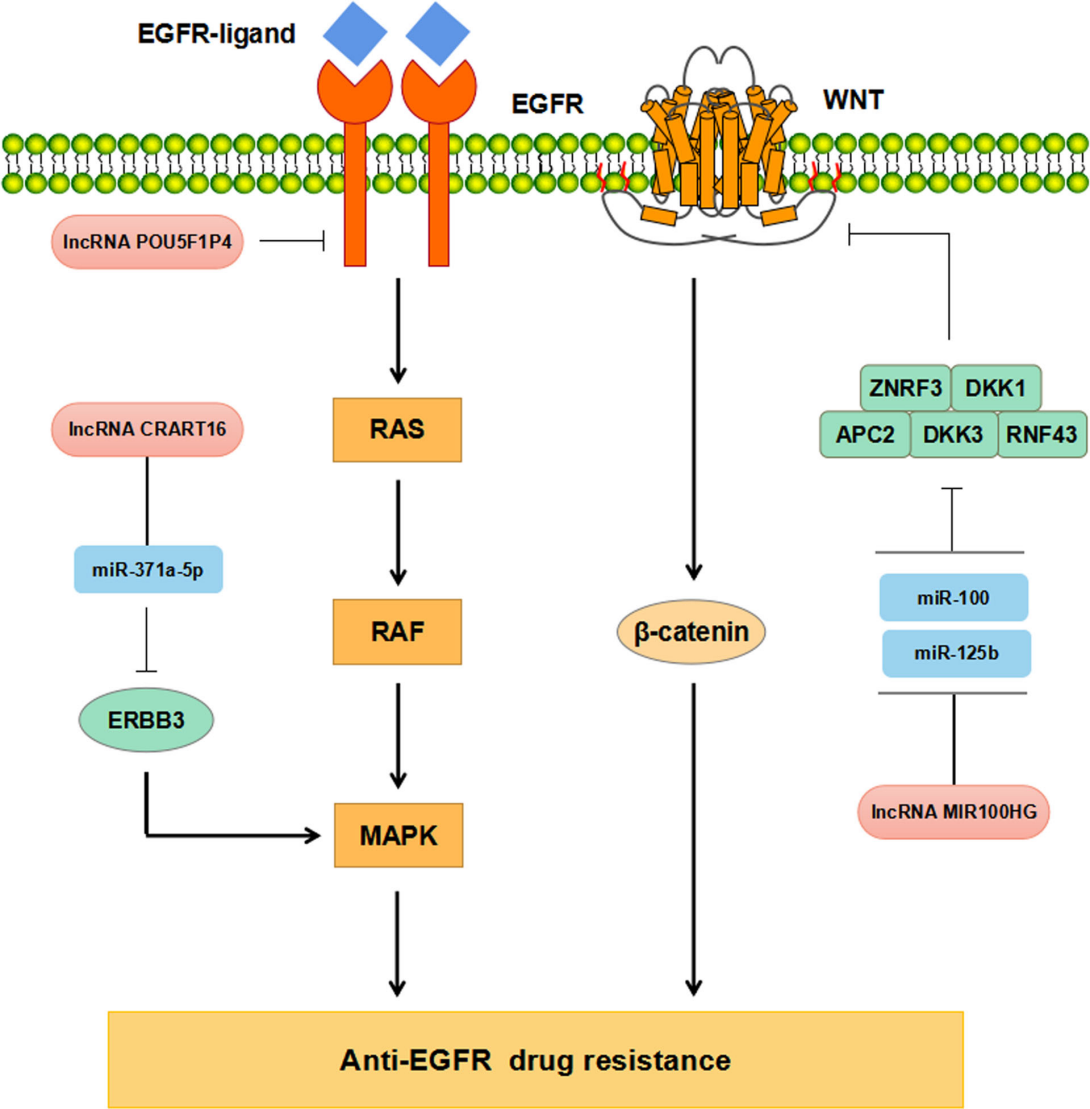
Mechanisms of lncRNAs regulating anti-EGFR drug resistance in CRC.

Increasing evidence has supported that lncRNAs participate in regulating CRC resistance to anti-EGFR McAbs through multiple signaling pathways (**Figure 3**). The study by Peng et al. has found that down-regulation of POU5F1P4 in cetuximab-sensitive CRC cells can reduce their sensitivity in mCRC (86). LNC00973 and several other lncRNAs may be involved in cetuximab resistance by regulating glucose metabolism (87). Down-regulation of LNC00973 can improve cetuximab resistance in drug-resistant CRC cells (87). Lu et al. have elaborated that the overexpression of lncRNA MIR100HG-derived miR-100 and miR-125b promotes cetuximab resistance through Wnt/β-catenin pathway in CRC (50) (**Tables 1** **,** **2**). Recent studies have reported that lncRNA CRART16 is up-regulated in CRC cells with secondary cetuximab-resistance. CRART16 contributes to cetuximab resistance in CRC by up-regulating ERBB3 through miR-371a-5p/MAPK signaling pathway (88). LncRNA HCG18 promotes cell proliferation, migration, and cetuximab resistance in CRC by up-regulating PD-L1 and down-regulating CD8^+^ T lymphocytes *via* sponging miR-20b-5p (89). Besides, the study by Yang et al. has shown the evidence that up-regulation of UCA1 in cetuximab-resistant CRC cells and the produced exosomes (90). Moreover, exosomal UCA1 is observed to cause drug resistance in cetuximab-sensitive CRC cells (90). Due to its non-invasive and relatively stable content in serum, exosomal UCA1 is hopefully used as a new biomarker for CRC in the future (**Table 2**).

**Table 2:** LncRNAs involved in anti-EGFR drugs resistance in CRC.

LncRNAs	Expression	Targets/Pathways	Drugs	References
POU5F1P4	Down-regulated	EGFR pathway	Cetuximab	(86)
LNC00973	Up-regulated	/	Cetuximab	(87)
MIR100HG	Up-regulated	MiR100/DKK1, ZNRF3/Wnt/β-catenin pathway, MiR-125b/ZNRF3, RNF43, DKK3, APC2/Wnt/β-catenin pathway	Cetuximab	(50)
CRART16	Up-regulated	MiR-371a-5p/ERBB3/MAPK pathway	Cetuximab	(88)
HCG18	Up-regulated	MiR-20b-5p/PD-L1	Cetuximab	(89)
UCA1	Up-regulated	/	Cetuximab	(90)

/, unmentioned in the reference.

Accumulated studies have suggested lncRNAs have been elucidated to serve as ceRNAs by sponging miRNAs, which subsequently regulates miRNAs-mediated anti-EGFR therapy resistance in CRC. In addition, lncRNAs play vital roles in CRC progression, metastasis, and drug resistance. These findings provide therapeutic targets and potential prognostic markers for CRC with regard to lncRNAs. Future studies are warranted to reveal the specific mechanism of lncRNAs involved in CRC progression, metastasis, and drug resistance.

## CircRNAs

### Biological Functions of CircRNAs

CircRNAs are novel covalently closed circular single-stranded ncRNAs discovered in recent years, mainly formed by exon reverse splicing of pre-mRNA. According to the sequence origin, circRNAs are grouped into exonic circRNAs, circular intronic RNAs, and exon-intron circRNAs (91). They exist stably in plasma, serum, saliva, and other body fluids, and are widely expressed in various types of cells with cell- and tissue-specificity (92, 93). Acting as ceRNAs, circRNAs can competitively bind with miRNAs and regulate gene expression *via* interacting with miRNAs or RNA-binding proteins (RBPs). They exert essential effects on the progression of multiple diseases including cancer (17, 94–99).

### CircRNAs Regulate Drug Resistance in CRC

**Figure 4:**
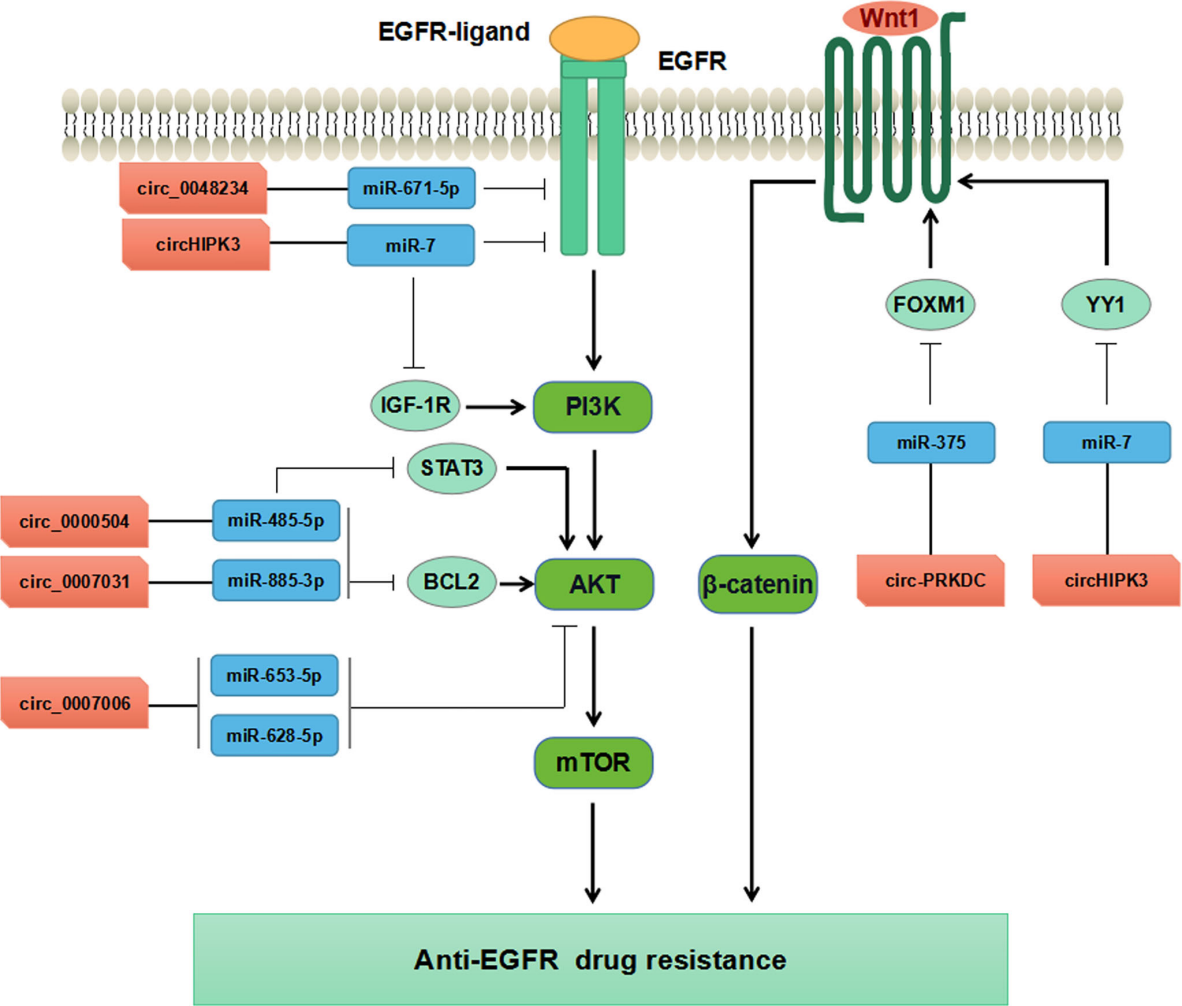
Mechanisms of circRNAs regulating CRC drug resistance by modulating AKT, EGFR, and WNT pathways.

Increasing evidence has supported that circRNAs participate in regulating tumorigenesis and drug resistance of CRC (100, 101). Chen et al. have found that circ-PRKDC acted as a miR-375 sponge and targeted FOXM1, and enhanced CRC cells resistance to 5-fluorouracil (5-FU) through the Wnt/β-catenin signaling pathway (102) (**Table 3**, **Figure 4**). CircRNAs of circ_0007031, circ_0007006, and circ_0000504 have been found to modulate 5-FU resistance of CRC cells by regulating AKT3 *via* the AKT signaling pathway, while circ_0048234 can sponge miR-671-5p in 5-FU-resistant CRC cells *via* the EGFR signaling pathway (103) (**Table 3**, **Figure 4**). ATP-binding cassette (ABC) transporters, such as ABCB1, ABCC1, and ABCG2, have been reported to play crucial roles in CRC drug resistance by increasing drug efflux out of cancer cells (105). Inhibition expression of ABC transporters is an effective approach to reverse drug resistance in cancer cells (105, 106). A number of ncRNAs have been demonstrated to be involved in regulating ABC transporters in drug-resistant cancer cells by regulating EGFR and its downstream signaling pathways (107, 108). Circ_0007031 has been documented to induce 5-FU resistance by modulating the expression of ABC transporter ABCC5 through miR-133b/ABCC5 axis in CRC (100). MiR-7 functions as a regulator of anti-EGFR therapy resistance in CRC. It has been shown that ciRS-7 regulated CRC cell growth and invasion by sponging miR-7 and upregulating EGFR and IGF-1R expression (109). Similarly, CiRS-7 can function as ceRNA for miR-7 to activate EGFR/RAF1/MAPK pathway in CRC (110). The study by Zeng et al. has reported circHIPK3 sponged miR-7 to upregulate the expression of several oncogenes, such as FAK, IGF1R, EGFR, and YY1, through the PI3K/AKT and MEK/ERK signaling pathways that contributing to drug resistance in CRC (104). Additionally, inhibition of circHIPK3 can reverse the resistance to cetuximab by targeting miR-7 in CRC cells (104) (**Figure 4**). All these findings have provided novel insights into the understanding of drug resistance mechanisms regarding circRNAs. Nevertheless, more studies are warranted to estimating the involvement and mechanism of circRNAs in regulating the resistance to anti-EGFR therapy in CRC.

**Table 3:** CircRNAs involved in drugs resistance in CRC.

CircRNAs	Expression	Targets/Pathways	Drugs	References
Circ-PRKDC	Up-regulated	MiR-375/FOXM1/Wnt/β-catenin pathway	5-FU	(102)
Circ_0007031	Up-regulated	MiR-885-3p/BCL2/AKT pathway	5-FU	(103)
		MiR-133b/ABCC5 axis		(100)
Circ_0007006	Up-regulated	MiR-653-5p, miR-628-5p/AKT pathway	5-FU	(103)
Circ_0000504	Up-regulated	MiR-485-5P/STAT3, BCL2/AKT pathway	5-FU	(103)
Circ_0048234	Down-regulated	MiR-671-5p/EGFR pathway	5-FU	(103)
CircHIPK3	Up-regulated	MiR-7/IGF-1R/PI3K/AKT pathway, MiR-7/EGFR/MEK/ERK pathway, MiR-7/YY1/Wnt pathway	Cetuximab	(104)

## Perspectives

Drug resistance remains a major challenge for CRC treatment. The mechanisms underlying CRC resistance to anti-EGFR therapy are complicated. Increasing studies have shown that ncRNAs play crucial roles in regulating the resistance to anti-EGFR therapy in CRC, primarily including miRNAs, lncRNAs and circRNAs, which have been identified as either oncogenes or tumor suppressors (111). Currently available studies have supported ncRNAs participate in modulating anti-EGFR drug resistance based on miRNAs-mRNAs, lncRNAs-miRNAs-mRNAs, or circRNAs-miRNAs-mRNAs regulatory networks through the EGFR signaling pathway, RAS signaling pathway, and PI3K/AKT signaling pathway. Accordingly, ncRNAs may function as novel biomarkers in predicting the efficacy and resistance of anti-EGFR therapy in CRC. Nevertheless, the molecular mechanisms of ncRNAs involved in anti-EGFR therapy resistance still warrant to be further elucidated in CRC. Further studies need to focus on investigating new therapeutic strategies based on ncRNAs regulatory networks combing with anti-EGFR targeted therapy in CRC.

## Author Contributions

SY, DX, and JC wrote the draft and revised it. JC, XF, ZS, LG, WD, HL, XY, JD, LLZ, and LZ collected the data and designed the tables and figures. All authors read and approved the submitted version.

## Funding

This work is supported by funds from the National Natural Science Foundation, China (82003042, 81870237, 82171790, 32000075) and Natural Science Foundation, Shandong Province (ZR2020KC001, ZR2019QH012, ZR2020QC012).

## Conflict of Interest

The authors declare that the research was conducted in the absence of any commercial or financial relationships that could be construed as a potential conflict of interest.

## Publisher’s Note

All claims expressed in this article are solely those of the authors and do not necessarily represent those of their affiliated organizations, or those of the publisher, the editors and the reviewers. Any product that may be evaluated in this article, or claim that may be made by its manufacturer, is not guaranteed or endorsed by the publisher.

## References

[1] Sung H Ferlay J Siegel RL Laversanne M Soerjomataram I Jemal A. Global Cancer Statistics 2020: GLOBOCAN Estimates of Incidence and Mortality Worldwide for 36 Cancers in 185 Countries. CA Cancer J Clin (2021) 71:209–49. doi: 10.3322/caac.21660 33538338

[2] Wang H. MicroRNAs and Apoptosis in Colorectal Cancer. Int J Mol Sci (2020) 21:5353. doi: 10.3390/ijms21155353 PMC7432330 32731413

[3] Pei X Liu Y Sun L Zhang J Fang Y Liao X. Outcome of Molecular Targeted Agents Plus Chemotherapy for Second-Line Therapy of Metastatic Colorectal Cancer: A Meta-Analysis of Randomized Trials. Clin Colorectal Cancer (2016) 15:e149–e56. doi: 10.1016/j.clcc.2016.03.005 27155750

[4] Fakih MG. Metastatic Colorectal Cancer: Current State and Future Directions. J Clin Oncol (2015) 33:1809–24. doi: 10.1200/JCO.2014.59.7633 25918280

[5] Zhao B Wang L Qiu H Zhang M Sun L Peng P. Mechanisms of Resistance to Anti-EGFR Therapy in Colorectal Cancer. Oncotarget (2017) 8:3980–4000. doi: 10.18632/oncotarget.14012 28002810 PMC5354808

[6] Amado RG Wolf M Peeters M Van Cutsem E Siena S Freeman DJ. Wild-Type KRAS is Required for Panitumumab Efficacy in Patients With Metastatic Colorectal Cancer. J Clin Oncol (2008) 26:1626–34. doi: 10.1200/JCO.2007.14.7116 18316791

[7] Schirripa M Lenz HJ. Colorectal Cancer: Overcoming Resistance to Anti-EGFR Therapy - Where do We Stand? Nat Rev Gastroenterol Hepatol (2016) 13:258–9. doi: 10.1038/nrgastro.2016.52 PMC7493690 27006256

[8] Dienstmann R Salazar R Tabernero J. Overcoming Resistance to Anti-EGFR Therapy in Colorectal Cancer. Am Soc Clin Oncol Educ Book (2015) 35:e149–56. doi: 10.14694/EdBook_AM.2015.35.e149 25993166

[9] Bianco R Gelardi T Damiano V Ciardiello F Tortora G. Rational Bases for the Development of EGFR Inhibitors for Cancer Treatment. Int J Biochem Cell Biol (2007) 39:1416–31. doi: 10.1016/j.biocel.2007.05.008 17596994

[10] Lan SH Wu SY Zuchini R Lin XZ Su IJ Tsai TF. Autophagy Suppresses Tumorigenesis of Hepatitis B Virus-Associated Hepatocellular Carcinoma Through Degradation of microRNA-224. Hepatol (2014) 59:505–17. doi: 10.1002/hep.26659 PMC4298796 23913306

[11] Zhou K Liu M Cao Y. New Insight Into microRNA Functions in Cancer: Oncogene-microRNA-Tumor Suppressor Gene Network. Front Mol Biosci (2017) 4:46. doi: 10.3389/fmolb.2017.00046 28736730 PMC5500619

[12] Yue QY Zhang Y. Effects of Linc00460 on Cell Migration and Invasion Through Regulating Epithelial-Mesenchymal Transition (EMT) in non-Small Cell Lung Cancer. Eur Rev Med Pharmacol Sci (2018) 22:1003–10. 10.26355/eurrev_201802_14382 29509248

[13] Song TF Huang LW Yuan Y Wang HQ He HP Ma WJ. LncRNA MALAT1 Regulates Smooth Muscle Cell Phenotype Switch *via* Activation of Autophagy. Oncotarget (2018) 9:4411–26. doi: 10.18632/oncotarget.23230 PMC5796983 29435112

[14] Li YF Pei FL Cao MZ. CircRNA_101951 Promotes Migration and Invasion of Colorectal Cancer Cells by Regulating the KIF3A-Mediated EMT Pathway. Exp Ther Med (2020) 19:3355–61. doi: 10.3892/etm.2020.8600 PMC7132243 32266033

[15] Li XN Wang ZJ Ye CX Zhao BC Huang XX Yang L. Circular RNA circVAPA is Up-Regulated and Exerts Oncogenic Properties by Sponging miR-101 in Colorectal Cancer. BioMed Pharmacother (2019) 112:108611. doi: 10.1016/j.biopha.2019.108611 30797148

[16] Shabaninejad Z Vafadar A Movahedpour A Ghasemi Y Namdar A Fathizadeh H. Circular RNAs in Cancer: New Insights Into Functions and Implications in Ovarian Cancer. J Ovarian Res (2019) 12:84. doi: 10.1186/s13048-019-0558-5 31481095 PMC6724287

[17] Zeng K Wang S. Circular RNAs: The Crucial Regulatory Molecules in Colorectal Cancer. Pathol Res Pract (2020) 216:152861. doi: 10.1016/j.prp.2020.152861 32061452

[18] Slattery ML Mullany LE Sakoda LC Wolff RK Samowitz WS Herrick JS. Dysregulated Genes and miRNAs in the Apoptosis Pathway in Colorectal Cancer Patients. Apoptosis (2018) 23:237–50. doi: 10.1007/s10495-018-1451-1 PMC5856858 29516317

[19] Kopp F Mendell JT. Functional Classification and Experimental Dissection of Long Noncoding RNAs. Cell (2018) 172:393–407. doi: 10.1016/j.cell.2018.01.011 29373828 PMC5978744

[20] Xie X Tang B Xiao YF Xie R Li BS Dong H. Long non-Coding RNAs in Colorectal Cancer. Oncotarget (2016) 7:5226–39. doi: 10.18632/oncotarget.6446 PMC4868682 26637808

[21] Chen W Cai G Liao Z Lin K Li G Li Y. miRNA-766 Induces Apoptosis of Human Colon Cancer Cells Through the P53/Bax Signaling Pathway by MDM4. Exp Ther Med (2019) 17:4100–8. doi: 10.3892/etm.2019.7436 PMC6468453 31007746

[22] O'brien J Hayder H Zayed Y Peng C. Overview of MicroRNA Biogenesis, Mechanisms of Actions, and Circulation. Front Endocrinol (Lausanne) (2018) 9:402. doi: 10.3389/fendo.2018.00402 30123182 PMC6085463

[23] Ha M Kim VN. Regulation of microRNA Biogenesis. Nat Rev Mol Cell Biol (2014) 15:509–24. doi: 10.1038/nrm3838 25027649

[24] Rizzuti M Filosa G Melzi V Calandriello L Dioni L Bollati V. MicroRNA Expression Analysis Identifies a Subset of Downregulated miRNAs in ALS Motor Neuron Progenitors. Sci Rep (2018) 8:10105. doi: 10.1038/s41598-018-28366-1 29973608 PMC6031650

[25] Rupaimoole R Slack FJ. MicroRNA Therapeutics: Towards a New Era for the Management of Cancer and Other Diseases. Nat Rev Drug Discov (2017) 16:203–22. doi: 10.1038/nrd.2016.246 28209991

[26] Liu SQ Jiang S Li C Zhang B Li QJ. miR-17-92 Cluster Targets Phosphatase and Tensin Homology and Ikaros Family Zinc Finger 4 to Promote TH17-Mediated Inflammation. J Biol Chem (2014) 289:12446–56. doi: 10.1074/jbc.M114.550723 PMC4007439 24644282

[27] Dragomir MP Kopetz S Ajani JA Calin GA. Non-Coding RNAs in GI Cancers: From Cancer Hallmarks to Clinical Utility. Gut (2020) 69:748–63. doi: 10.1136/gutjnl-2019-318279 32034004

[28] Goh SY Chao YX Dheen ST Tan E-K Tay SS-W. Role of MicroRNAs in Parkinson’s Disease. Int J Mol Sci (2019) 20:5649. doi: 10.3390/ijms20225649 PMC6888719 31718095

[29] Mayr C Beyreis M Wagner A Pichler M Neureiter D Kiesslich T. Deregulated MicroRNAs in Biliary Tract Cancer: Functional Targets and Potential Biomarkers. BioMed Res Int (2016) 2016:4805270. doi: 10.1155/2016/4805270 27957497 PMC5120202

[30] Wen D Danquah M Chaudhary AK Mahato RI. Small Molecules Targeting microRNA for Cancer Therapy: Promises and Obstacles. J Control Release (2015) 219:237–47. doi: 10.1016/j.jconrel.2015.08.011 PMC4749151 26256260

[31] Fanini F Fabbri M. MicroRNAs and Cancer Resistance: A New Molecular Plot. Clin Pharmacol Ther (2016) 99:485–93. doi: 10.1002/cpt.353 26875151

[32] Maleki M Golchin A Javadi S Khelghati N Morovat P Asemi Z. Role of Exosomal miRNA in Chemotherapy Resistance of Colorectal Cancer: A Systematic Review. Chem Biol Drug Des (2021). doi: 10.1111/cbdd.13947 34480511

[33] Valastyan S Reinhardt F Benaich N Calogrias D Szasz AM Wang ZC. A Pleiotropically Acting microRNA, miR-31, Inhibits Breast Cancer Metastasis. Cell (2009) 137:1032–46. doi: 10.1016/j.cell.2009.03.047 PMC2766609 19524507

[34] Liu X Sempere LF Ouyang H Memoli VA Andrew AS Luo Y. MicroRNA-31 Functions as an Oncogenic microRNA in Mouse and Human Lung Cancer Cells by Repressing Specific Tumor Suppressors. J Clin Invest (2010) 120:1298–309. doi: 10.1172/JCI39566 PMC2846041 20237410

[35] Sun D Yu F Ma Y Zhao R Chen X Zhu J. MicroRNA-31 Activates the RAS Pathway and Functions as an Oncogenic MicroRNA in Human Colorectal Cancer by Repressing RAS P21 GTPase Activating Protein 1 (RASA1). J Biol Chem (2013) 288:9508–18. doi: 10.1074/jbc.M112.367763 PMC3611019 23322774

[36] Chen T Yao LQ Shi Q Ren Z Ye LC Xu JM. MicroRNA-31 Contributes to Colorectal Cancer Development by Targeting Factor Inhibiting HIF-1alpha (FIH-1). Cancer Biol Ther (2014) 15:516–23. doi: 10.4161/cbt.28017 PMC4026074 24521875

[37] Sur D Cainap C Burz C Havasi A Chis IC Vlad C. The Role of miRNA -31-3p and miR-31-5p in the Anti-EGFR Treatment Efficacy of Wild-Type K-RAS Metastatic Colorectal Cancer. Is it Really the Next Best Thing in miRNAs? J BUON (2019) 24:1739–46. 31786833

[38] Mekenkamp LJ Tol J Dijkstra JR De Krijger I Vink-Borger ME Van Vliet S. Beyond KRAS Mutation Status: Influence of KRAS Copy Number Status and microRNAs on Clinical Outcome to Cetuximab in Metastatic Colorectal Cancer Patients. BMC Cancer (2012) 12:292. doi: 10.1186/1471-2407-12-292 22804917 PMC3508829

[39] Igarashi H Kurihara H Mitsuhashi K Ito M Okuda H Kanno S. Association of MicroRNA-31-5p With Clinical Efficacy of Anti-EGFR Therapy in Patients With Metastatic Colorectal Cancer. Ann Surg Oncol (2015) 22:2640–8. doi: 10.1245/s10434-014-4264-7 25472647

[40] Zhou J Lv L Lin C Hu G Guo Y Wu M. Combinational Treatment With microRNA−133b and Cetuximab has Increased Inhibitory Effects on the Growth and Invasion of Colorectal Cancer Cells by Regulating EGFR. Mol Med Rep (2015) 12:5407–14. doi: 10.3892/mmr.2015.4046 26151111

[41] Suto T Yokobori T Yajima R Morita H Fujii T Yamaguchi S. MicroRNA-7 Expression in Colorectal Cancer is Associated With Poor Prognosis and Regulates Cetuximab Sensitivity *via* EGFR Regulation. Carcinogenesis (2015) 36:338–45. doi: 10.1093/carcin/bgu242 25503932

[42] Sun L Fang Y Wang X Han Y Du F Li C. miR-302a Inhibits Metastasis and Cetuximab Resistance in Colorectal Cancer by Targeting NFIB and CD44. Theranostics (2019) 9:8409–25. doi: 10.7150/thno.36605 PMC6857048 31754405

[43] Akao Y Kumazaki M Shinohara H Sugito N Kuranaga Y Tsujino T. Impairment of K-Ras Signaling Networks and Increased Efficacy of Epidermal Growth Factor Receptor Inhibitors by a Novel Synthetic miR-143. Cancer Sci (2018) 109:1455–67. doi: 10.1111/cas.13559 PMC5980131 29498789

[44] Gomes SE Simoes AE Pereira DM Castro RE Rodrigues CM Borralho PM. miR-143 or miR-145 Overexpression Increases Cetuximab-Mediated Antibody-Dependent Cellular Cytotoxicity in Human Colon Cancer Cells. Oncotarget (2016) 7:9368–87. doi: 10.18632/oncotarget.7010 PMC4891046 26824186

[45] Strippoli A Cocomazzi A Basso M Cenci T Ricci R Pierconti F. C-MYC Expression Is a Possible Keystone in the Colorectal Cancer Resistance to EGFR Inhibitors. Cancers (Basel) (2020) 12:638. doi: 10.3390/cancers12030638 PMC7139615 32164324

[46] Chu YC Tsai TY Yadav VK Deng L Huang CC Tzeng YM. 4-Acetyl-Antroquinonol B Improves the Sensitization of Cetuximab on Both Kras Mutant and Wild Type Colorectal Cancer by Modulating the Expression of Ras/Raf/miR-193a-3p Signaling Axis. Int J Mol Sci (2021) 22:7508. doi: 10.3390/ijms22147508 34299137 PMC8307961

[47] Hiraide S Takahashi M Yoshida Y Yamada H Komine K Ishioka C. Tumor Suppressor miR-193a-3p Enhances Efficacy of BRAF/MEK Inhibitors in BRAF-Mutated Colorectal Cancer. Cancer Sci (2021) 112:3856–70. doi: 10.1111/cas.15075 PMC8409311 34288281

[48] Weng WH Leung WH Pang YJ Hsu HH. Lauric Acid can Improve the Sensitization of Cetuximab in KRAS/BRAF Mutated Colorectal Cancer Cells by Retrievable microRNA-378 Expression. Oncol Rep (2016) 35:107–16. doi: 10.3892/or.2015.4336 26496897

[49] Weng WH Leung WH Pang YJ Kuo LW Hsu HH. EPA Significantly Improves Anti-EGFR Targeted Therapy by Regulating miR-378 Expression in Colorectal Cancer. Oncol Lett (2018) 16:6188–94. doi: 10.3892/ol.2018.9408 PMC6176383 30333883

[50] Lu Y Zhao X Liu Q Li C Graves-Deal R Cao Z. lncRNA MIR100HG-Derived miR-100 and miR-125b Mediate Cetuximab Resistance *via* Wnt/beta-Catenin Signaling. Nat Med (2017) 23:1331–41. doi: 10.1038/nm.4424 PMC5961502 29035371

[51] Mussnich P Rosa R Bianco R Fusco A D'angelo D. MiR-199a-5p and miR-375 Affect Colon Cancer Cell Sensitivity to Cetuximab by Targeting PHLPP1. Expert Opin Ther Targets (2015) 19:1017–26. doi: 10.1517/14728222.2015.1057569 26107137

[52] Alam KJ Mo JS Han SH Park WC Kim HS Yun KJ. MicroRNA 375 Regulates Proliferation and Migration of Colon Cancer Cells by Suppressing the CTGF-EGFR Signaling Pathway. Int J Cancer (2017) 141:1614–29. doi: 10.1002/ijc.30861 28670764

[53] Angius A Pira G Scanu AM Uva P Sotgiu G Saderi L. MicroRNA-425-5p Expression Affects BRAF/RAS/MAPK Pathways In Colorectal Cancers. Int J Med Sci (2019) 16:1480–91. doi: 10.7150/ijms.35269 PMC6818206 31673240

[54] Yue B Cai D Liu C Fang C Yan D. Linc00152 Functions as a Competing Endogenous RNA to Confer Oxaliplatin Resistance and Holds Prognostic Values in Colon Cancer. Mol Ther (2016) 24:2064–77. doi: 10.1038/mt.2016.180 PMC5167786 27633443

[55] Pekow J Meckel K Dougherty U Huang Y Chen X Almoghrabi A. miR-193a-3p is a Key Tumor Suppressor in Ulcerative Colitis-Associated Colon Cancer and Promotes Carcinogenesis Through Upregulation of IL17RD. Clin Cancer Res (2017) 23:5281–91. doi: 10.1158/1078-0432.CCR-17-0171 PMC5581687 28600480

[56] Takahashi H Takahashi M Ohnuma S Unno M Yoshino Y Ouchi K. microRNA-193a-3p is Specifically Down-Regulated and Acts as a Tumor Suppressor in BRAF-Mutated Colorectal Cancer. BMC Cancer (2017) 17:723. doi: 10.1186/s12885-017-3739-x 29115941 PMC5678600

[57] Liang L Gao C Li Y Sun M Xu J Li H. miR-125a-3p/FUT5-FUT6 Axis Mediates Colorectal Cancer Cell Proliferation, Migration, Invasion and Pathological Angiogenesis *via* PI3K-Akt Pathway. Cell Death Dis (2017) 8:e2968. doi: 10.1038/cddis.2017.352 28771224 PMC5596543

[58] Mao Q Quan T Luo B Guo X Liu L Zheng Q. MiR-375 Targets KLF4 and Impacts the Proliferation of Colorectal Carcinoma. Tumour Biol (2016) 37:463–71. doi: 10.1007/s13277-015-3809-0 26224477

[59] Tian L Chen M He Q Yan Q Zhai C. MicroRNA199a5p Suppresses Cell Proliferation, Migration and Invasion by Targeting ITGA3 in Colorectal Cancer. Mol Med Rep (2020) 22:2307–17. doi: 10.3892/mmr.2020.11323 PMC7411411 32705201

[60] Xu L Wen T Liu Z Xu F Yang L Liu J. MicroRNA-375 Suppresses Human Colorectal Cancer Metastasis by Targeting Frizzled 8. Oncotarget (2016) 7:40644–56. doi: 10.18632/oncotarget.9811 PMC5130033 27276676

[61] Wang Y Tang Q Li M Jiang S Wang X. MicroRNA-375 Inhibits Colorectal Cancer Growth by Targeting PIK3CA. Biochem Biophys Res Commun (2014) 444:199–204. doi: 10.1016/j.bbrc.2014.01.028 24440701

[62] Chao CC Wu PH Huang HC Chung HY Chou YC Cai BH. Downregulation of miR-199a/B-5p is Associated With GCNT2 Induction Upon Epithelial-Mesenchymal Transition in Colon Cancer. FEBS Lett (2017) 591:1902–17. doi: 10.1002/1873-3468.12685 28542779

[63] Junttila MR De Sauvage FJ. Influence of Tumour Micro-Environment Heterogeneity on Therapeutic Response. Nat (2013) 501:346–54. doi: 10.1038/nature12626 24048067

[64] Altorki NK Markowitz GJ Gao D Port JL Saxena A Stiles B. The Lung Microenvironment: An Important Regulator of Tumour Growth and Metastasis. Nat Rev Cancer (2019) 19:9–31. doi: 10.1038/s41568-018-0081-9 30532012 PMC6749995

[65] Lee SS Cheah YK. The Interplay Between MicroRNAs and Cellular Components of Tumour Microenvironment (TME) on Non-Small-Cell Lung Cancer (NSCLC) Progression. J Immunol Res (2019) 2019:3046379. doi: 10.1155/2019/3046379 30944831 PMC6421779

[66] Meng F Yang M Chen Y Chen W Wang W. miR-34a Induces Immunosuppression in Colorectal Carcinoma Through Modulating a SIRT1/NF-Kappab/B7-H3/TNF-Alpha Axis. Cancer Immunol Immunother (2021) 70:2247–59. doi: 10.1007/s00262-021-02862-2 PMC10991903 33492448

[67] Zheng J Yang T Gao S Cheng M Shao Y Xi Y. miR-148a-3p Silences the CANX/MHC-I Pathway and Impairs CD8(+) T Cell-Mediated Immune Attack in Colorectal Cancer. FASEB J (2021) 35:e21776. doi: 10.1096/fj.202100235R 34324740

[68] Lou Q Liu R Yang X Li W Huang L Wei L. miR-448 Targets IDO1 and Regulates CD8(+) T Cell Response in Human Colon Cancer. J Immunother Cancer (2019) 7:210. doi: 10.1186/s40425-019-0691-0 31391111 PMC6686234

[69] Trivedi S Srivastava RM Concha-Benavente F Ferrone S Garcia-Bates TM Li J. Anti-EGFR Targeted Monoclonal Antibody Isotype Influences Antitumor Cellular Immunity in Head and Neck Cancer Patients. Clin Cancer Res (2016) 22:5229–37. doi: 10.1158/1078-0432.CCR-15-2971 PMC5093040 27217441

[70] Trotta AM Ottaiano A Romano C Nasti G Nappi A De Divitiis C. Prospective Evaluation of Cetuximab-Mediated Antibody-Dependent Cell Cytotoxicity in Metastatic Colorectal Cancer Patients Predicts Treatment Efficacy. Cancer Immunol Res (2016) 4:366–74. doi: 10.1158/2326-6066.CIR-15-0184 26817995

[71] Pecci F Cantini L Bittoni A Lenci E Lupi A Crocetti S. Beyond Microsatellite Instability: Evolving Strategies Integrating Immunotherapy for Microsatellite Stable Colorectal Cancer. Curr Treat Options Oncol (2021) 22:69. doi: 10.1007/s11864-021-00870-z 34110510 PMC8192371

[72] Bourhis J Stein A Paul De Boer J Van Den Eynde M Gold KA Stintzing S. Avelumab and Cetuximab as a Therapeutic Combination: An Overview of Scientific Rationale and Current Clinical Trials in Cancer. Cancer Treat Rev (2021) 97:102172. doi: 10.1016/j.ctrv.2021.102172 33989949

[73] Hombach S Kretz M. Non-Coding RNAs: Classification, Biology and Functioning. Adv Exp Med Biol (2016) 937:3–17. doi: 10.1007/978-3-319-42059-2_1 27573892

[74] Ponting CP Oliver PL Reik W. Evolution and Functions of Long Noncoding RNAs. Cell (2009) 136:629–41. doi: 10.1016/j.cell.2009.02.006 19239885

[75] Saha P Verma S Pathak RU Mishra RK. Long Noncoding RNAs in Mammalian Development and Diseases. Adv Exp Med Biol (2017) 1008:155–98. doi: 10.1007/978-981-10-5203-3_6 28815540

[76] Luo J Qu J Wu DK Lu ZL Sun YS Qu Q. Long non-Coding RNAs: A Rising Biotarget in Colorectal Cancer. Oncotarget (2017) 8:22187–202. doi: 10.18632/oncotarget.14728 PMC5400657 28108736

[77] Congrains A Kamide K Oguro R Yasuda O Miyata K Yamamoto E. Genetic Variants at the 9p21 Locus Contribute to Atherosclerosis Through Modulation of ANRIL and CDKN2A/B. Atherosclerosis (2012) 220:449–55. doi: 10.1016/j.atherosclerosis.2011.11.017 22178423

[78] Johnson R. Long non-Coding RNAs in Huntington's Disease Neurodegeneration. Neurobiol Dis (2012) 46:245–54. doi: 10.1016/j.nbd.2011.12.006 22202438

[79] Zhu Y Li B Liu Z Jiang L Wang G Lv M. Up-Regulation of lncRNA SNHG1 Indicates Poor Prognosis and Promotes Cell Proliferation and Metastasis of Colorectal Cancer by Activation of the Wnt/beta-Catenin Signaling Pathway. Oncotarget (2017) 8:111715–27. doi: 10.18632/oncotarget.22903 PMC5762354 29340086

[80] Liu T Han Z Li H Zhu Y Sun Z Zhu A. LncRNA DLEU1 Contributes to Colorectal Cancer Progression *via* Activation of KPNA3. Mol Cancer (2018) 17:118. doi: 10.1186/s12943-018-0873-2 30098595 PMC6087004

[81] Li M Wang Q Xue F Wu Y. lncRNA-CYTOR Works as an Oncogene Through the CYTOR/miR-3679-5p/MACC1 Axis in Colorectal Cancer. DNA Cell Biol (2019) 38:572–82. doi: 10.1089/dna.2018.4548 31144988

[82] Zhang XT Pan SX Wang AH Kong QY Jiang KT Yu ZB. Long Non-Coding RNA (lncRNA) X-Inactive Specific Transcript (XIST) Plays a Critical Role in Predicting Clinical Prognosis and Progression of Colorectal Cancer. Med Sci Monit (2019) 25:6429–35. doi: 10.12659/MSM.915329 PMC6724558 31452526

[83] Qian J Garg A Li F Shen Q Xiao K. lncRNA LUNAR1 Accelerates Colorectal Cancer Progression by Targeting the Mir4953p/MYCBP Axis. Int J Oncol (2020) 57:1157–68. doi: 10.3892/ijo.2020.5128 PMC7549538 33300052

[84] Ma Y Yang Y Wang F Moyer MP Wei Q Zhang P. Long non-Coding RNA CCAL Regulates Colorectal Cancer Progression by Activating Wnt/beta-Catenin Signalling Pathway *via* Suppression of Activator Protein 2alpha. Gut (2016) 65:1494–504. doi: 10.1136/gutjnl-2014-308392 25994219

[85] Wang H Guan Z He K Qian J Cao J Teng L. LncRNA UCA1 in Anti-Cancer Drug Resistance. Oncotarget (2017) 8:64638–50. doi: 10.18632/oncotarget.18344 PMC5610032 28969100

[86] Peng K Liu R Yu Y Liang L Yu S Xu X. Identification and Validation of Cetuximab Resistance Associated Long Noncoding RNA Biomarkers in Metastatic Colorectal Cancer. BioMed Pharmacother (2018) 97:1138–46. doi: 10.1016/j.biopha.2017.11.031 29136952

[87] Jing C Ma R Cao H Wang Z Liu S Chen D. Long Noncoding RNA and mRNA Profiling in Cetuximab-Resistant Colorectal Cancer Cells by RNA Sequencing Analysis. Cancer Med (2019) 8:1641–51. doi: 10.1002/cam4.2004 PMC6488152 30848094

[88] Zhang X Wen L Chen S Zhang J Ma Y Hu J. The Novel Long Noncoding RNA CRART16 Confers Cetuximab Resistance in Colorectal Cancer Cells by Enhancing ERBB3 Expression *via* miR-371a-5p. Cancer Cell Int (2020) 20:68. doi: 10.1186/s12935-020-1155-9 32158358 PMC7057486

[89] Xu YJ Zhao JM Ni XF Wang W Hu WW Wu CP. LncRNA HCG18 Suppresses CD8(+) T Cells to Confer Resistance to Cetuximab in Colorectal Cancer *via* miR-20b-5p/PD-L1 Axis. Epigenomics (2021) 13:1281–97. doi: 10.2217/epi-2021-0130 34523356

[90] Yang YN Zhang R Du JW Yuan HH Li YJ Wei XL. Predictive Role of UCA1-Containing Exosomes in Cetuximab-Resistant Colorectal Cancer. Cancer Cell Int (2018) 18:164. doi: 10.1186/s12935-018-0660-6 30377411 PMC6196422

[91] Chen LL Yang L. Regulation of circRNA Biogenesis. RNA Biol (2015) 12:381–8. doi: 10.1080/15476286.2015.1020271 PMC4615371 25746834

[92] Barrett SP Salzman J. Circular RNAs: Analysis, Expression and Potential Functions. Development (2016) 143:1838–47. doi: 10.1242/dev.128074 PMC4920157 27246710

[93] Su M Xiao Y Ma J Tang Y Tian B Zhang Y. Circular RNAs in Cancer: Emerging Functions in Hallmarks, Stemness, Resistance and Roles as Potential Biomarkers. Mol Cancer (2019) 18:90. doi: 10.1186/s12943-019-1002-6 30999909 PMC6471953

[94] Lukiw WJ. Circular RNA (circRNA) in Alzheimer's Disease (AD). Front Genet (2013) 4:307. doi: 10.3389/fgene.2013.00307 24427167 PMC3875874

[95] Liu Q Shuai M Xia Y. Knockdown of EBV-Encoded circRNA Circrpms1 Suppresses Nasopharyngeal Carcinoma Cell Proliferation and Metastasis Through Sponging Multiple miRNAs. Cancer Manag Res (2019) 11:8023–31. doi: 10.2147/CMAR.S218967 PMC6717849 31695488

[96] Li O Kang J Zhang JJ Wang J Hu LW Li L. Circle RNA FOXP1 Promotes Cell Proliferation in Lung Cancer by Regulating miR-185-5p/Wnt1 Signaling Pathway. Eur Rev Med Pharmacol Sci (2020) 24:6767–78. doi: 10.26355/eurrev_202006_21665 32633368

[97] Zhan W Liao X Chen Z Li L Tian T Yu L. Circular RNA Hsa_circRNA_103809 Promoted Hepatocellular Carcinoma Development by Regulating miR-377-3p/FGFR1/ERK Axis. J Cell Physiol (2020) 235:1733–45. doi: 10.1002/jcp.29092 31317555

[98] Zhang S Wang W Wu X Zhou X. Regulatory Roles of Circular RNAs in Coronary Artery Disease. Mol Ther Nucleic Acids (2020) 21:172–9. doi: 10.1016/j.omtn.2020.05.024 PMC7321795 32585625

[99] Zhao Y Zhong R Deng C Zhou Z. Circle RNA Circabcb10 Modulates PFN2 to Promote Breast Cancer Progression, as Well as Aggravate Radioresistance Through Facilitating Glycolytic Metabolism *Via* miR-223-3p. Cancer Biother Radiopharm (2021) 36:477–90. doi: 10.1089/cbr.2019.3389 32522014

[100] He X Ma J Zhang M Cui J Yang H. Circ_0007031 Enhances Tumor Progression and Promotes 5-Fluorouracil Resistance in Colorectal Cancer Through Regulating miR-133b/ABCC5 Axis. Cancer biomark (2020) 29:531–42. doi: 10.3233/cbm-200023 PMC12662547 32865180

[101] Liu N Jiang F. Chen Z. A Preliminary Study on the Pathogenesis of Colorectal Cancer by Constructing a Hsa-circRNA-0067835-miRNA-mRNA Regulatory Network. Onco Targets Ther (2021) 14:4645–58. doi: 10.2147/OTT.S319300 PMC8418363 34511934

[102] Chen H Pei L Xie P Guo G. Circ-PRKDC Contributes to 5-Fluorouracil Resistance of Colorectal Cancer Cells by Regulating miR-375/FOXM1 Axis and Wnt/β-Catenin Pathway. Onco Targets Ther (2020) 13:5939–53. doi: 10.2147/ott.S253468 PMC7320885 32606803

[103] Xiong W Ai YQ Li YF Ye Q Chen ZT Qin JY. Microarray Analysis of Circular RNA Expression Profile Associated With 5-Fluorouracil-Based Chemoradiation Resistance in Colorectal Cancer Cells. BioMed Res Int (2017) 2017:8421614. doi: 10.1155/2017/8421614 28656150 PMC5471554

[104] Zeng K Chen X Xu M Liu X Hu X Xu T. CircHIPK3 Promotes Colorectal Cancer Growth and Metastasis by Sponging miR-7. Cell Death Dis (2018) 9:417. doi: 10.1038/s41419-018-0454-8 29549306 PMC5856798

[105] Hu T Li Z Gao CY Cho CH. Mechanisms of Drug Resistance in Colon Cancer and its Therapeutic Strategies. World J Gastroenterol (2016) 22:6876–89. doi: 10.3748/wjg.v22.i30.6876 PMC4974586 27570424

[106] Gao Q Li XX Xu YM Zhang JZ Rong SD Qin YQ. IRE1alpha-Targeting Downregulates ABC Transporters and Overcomes Drug Resistance of Colon Cancer Cells. Cancer Lett (2020) 476:67–74. doi: 10.1016/j.canlet.2020.02.007 32061752

[107] Jiang L Wang P Sun YJ Wu YJ. Ivermectin Reverses the Drug Resistance in Cancer Cells Through EGFR/ERK/Akt/NF-kappaB Pathway. J Exp Clin Cancer Res (2019) 38:265. doi: 10.1186/s13046-019-1251-7 31215501 PMC6580523

[108] Wang Y Wang Y Qin Z Cai S Yu L Hu H. The Role of non-Coding RNAs in ABC Transporters Regulation and Their Clinical Implications of Multidrug Resistance in Cancer. Expert Opin Drug Metab Toxicol (2021) 17:291–306. doi: 10.1080/17425255.2021.1887139 33544643

[109] Tang W Ji M He G Yang L Niu Z Jian M. Silencing CDR1as Inhibits Colorectal Cancer Progression Through Regulating microRNA-7. Onco Targets Ther (2017) 10:2045–56. doi: 10.2147/OTT.S131597 PMC5391170 28435295

[110] Weng W Wei Q Toden S Yoshida K Nagasaka T Fujiwara T. Circular RNA ciRS-7-A Promising Prognostic Biomarker and a Potential Therapeutic Target in Colorectal Cancer. Clin Cancer Res (2017) 23:3918–28. doi: 10.1158/1078-0432.CCR-16-2541 PMC5511556 28174233

[111] Long F Lin Z Li L Ma M Lu Z Jing L. Comprehensive Landscape and Future Perspectives of Circular RNAs in Colorectal Cancer. Mol Cancer (2021) 20:26. doi: 10.1186/s12943-021-01318-6 33536039 PMC7856739

